# Non-stem cell-derived exosomes: a novel therapeutics for neurotrauma

**DOI:** 10.1186/s12951-024-02380-0

**Published:** 2024-03-12

**Authors:** Xinyu Nie, Tianyang Yuan, Tong Yu, Zhihe Yun, Tao Yu, Qinyi Liu

**Affiliations:** Department of Orthopaedic, The second hospital of Jilin University, Changchun, China

**Keywords:** Exosomes, Neurotrauma, Therapeutics, Tissue regeneration, Non-stem cell sources

## Abstract

Neurotrauma, encompassing traumatic brain injuries (TBI) and spinal cord injuries (SCI) impacts a significant portion of the global population. While spontaneous recovery post-TBI or SCI is possible, recent advancements in cell-based therapies aim to bolster these natural reparative mechanisms. Emerging research indicates that the beneficial outcomes of such therapies might be largely mediated by exosomes secreted from the administered cells. While stem cells have garnered much attention, exosomes derived from non-stem cells, including neurons, Schwann cells, microglia, and vascular endothelial cells, have shown notable therapeutic potential. These exosomes contribute to angiogenesis, neurogenesis, and axon remodeling, and display anti-inflammatory properties, marking them as promising agents for neurorestorative treatments. This review provides an in-depth exploration of the current methodologies, challenges, and future directions regarding the therapeutic role of non-stem cell-derived exosomes in neurotrauma.

## Introduction

Neurotrauma, such as traumatic brain injury (TBI) and spinal cord injury (SCI), remain leading contributors to the development of diseases and mortality rates associated with trauma [1, 2]. A study conducted by the global burden of disease collaborative group revealed that there were approximately 27.08 million new instances of TBI and 0.93 million new cases of SCI across the globe. Additionally, the worldwide prevalence of TBI and SCI has been estimated at 55.50 million and 27.04 million [1, 3, 4]. Given the increasing global prevalence of these injuries, it is of urgent importance to find innovative methods for treating neurotrauma.

### The Pathology of Neurotrauma

Understanding the intricate pathology of neurotrauma is pivotal, as it not only elucidates the mechanisms underpinning injury progression but also illuminates potential therapeutic targets for intervention. Neurotrauma’s pathology encompasses two primary stages: primary and secondary injuries. The primary injury is the direct result of trauma, leading to irreversible mechanical damage to the brain and spinal cord. This injury disrupts both the Blood-Brain Barrier (BBB) and Blood-Spinal Cord Barrier (BSCB), Such disruptions result in the release of inhibitory products, such as neuronal growth inhibitor protein A and chondroitin sulfate proteoglycans, which significantly impede axonal regeneration [5–7]. The secondary injury phase, which follows the initial trauma, is characterized by a more extensive disruption of the neural tissue. This phase extends from minutes after the initial injury to weeks or even longer and involves a complex interplay of factors such as inflammation, ischemia, oxidative stress, excitotoxicity, and apoptotic cell death [5, 7–9]. During this phase, cells such as macrophages, neutrophils, and T cells become active, releasing factors that promote inflammation, exacerbating the lesion and its associated tissue damage [10, 11]. At the same time, an imbalance exists between reactive oxygen species (ROS) and antioxidant mechanisms. While mitochondria normally generate ROS during oxidative phosphorylation, post-neurotraumatic mitochondrial impairment results in heightened ROS levels. This escalation in ROS contributes to oxidative harm and triggers apoptotic processes [12, 13]. These secondary injury processes exacerbate the damage initiated by the primary injury and significantly influence the extent of functional recovery and clinical outcomes.

Despite significant advancements in comprehending the underlying mechanisms of neurotrauma, individuals with moderate to severe injuries in this area continue to experience persistent neurological impairments.

### Current therapeutic strategies and their limitations

**Fig. 1:**
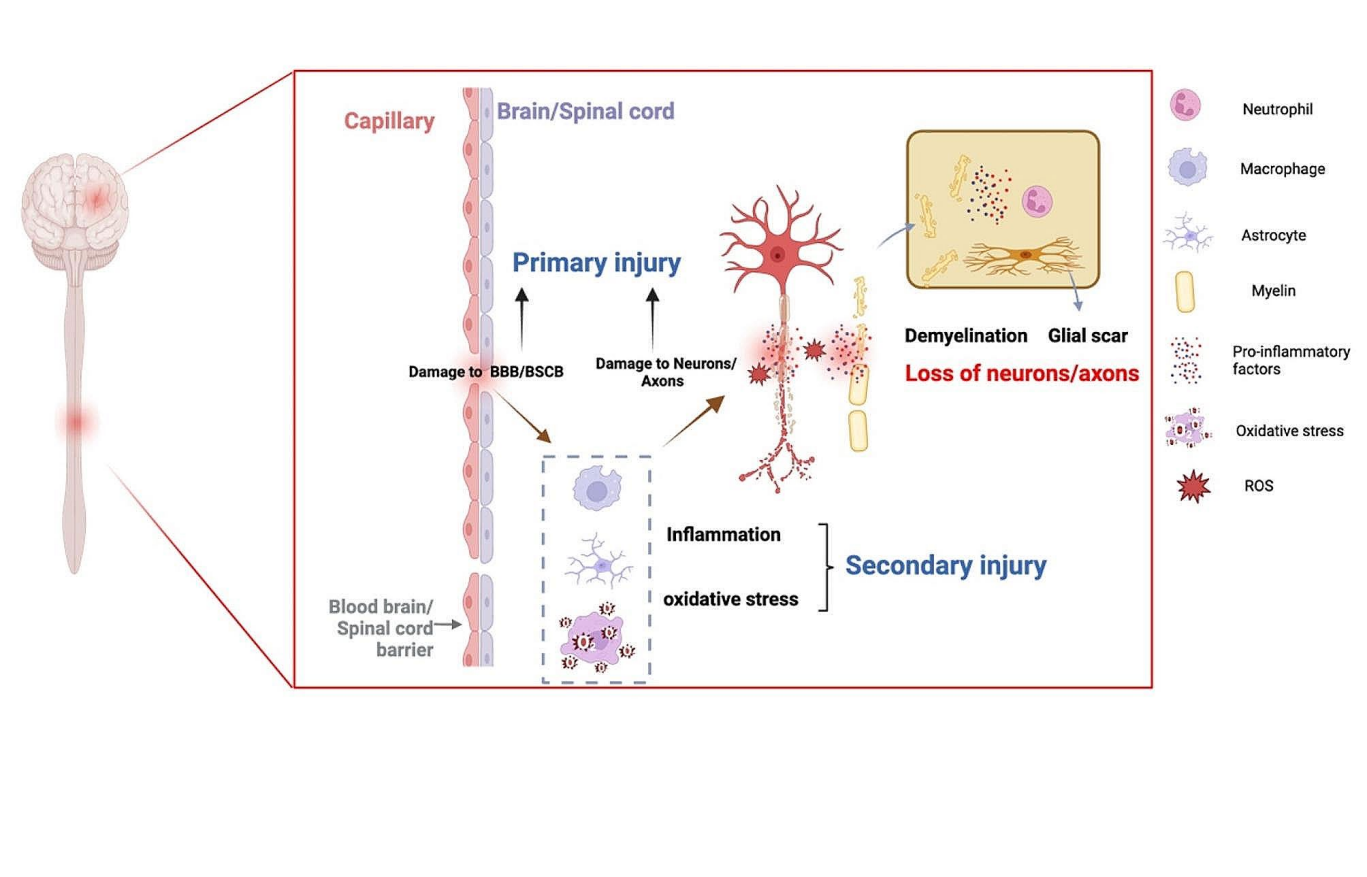
A range of common pathologic mechanisms following TBI and SCI: primary injury including demyelination and cyst formation. Secondary injury includes loss of neurons/axons, inflammation, and glial scar

Currently, two primary treatment strategies exist for neurotrauma. The initial approach focuses on protecting the surviving neurons and axons from further damage during the immediate aftermath of the injury. This protection can be achieved through measures such as prompt clinical decompression, or by targeting and reducing secondary inflammation. Specific treatments may include the administration of medications like high-dosage methylprednisolone, calcium channel blockers, and naloxone, along with techniques such as localized cryoprotection and synthetic hyperbaric blood flow [14–16]. The subsequent strategy emphasizes cellular therapy, aiming to assist in the repair and renewal of neural tissues during the injury’s chronic phase. This approach may incorporate surgical interventions, stem cell grafting, hyperbaric oxygen treatments, and other specialized techniques [17–20]. However, these approaches have limitations and often fall short of achieving the primary goals of anti-inflammatory action, Central Nervous System (CNS) damage replacement, and regenerative therapy for neurotrauma (Fig. 1).

### Therapeutic potential of exosomes

**Fig. 2:**
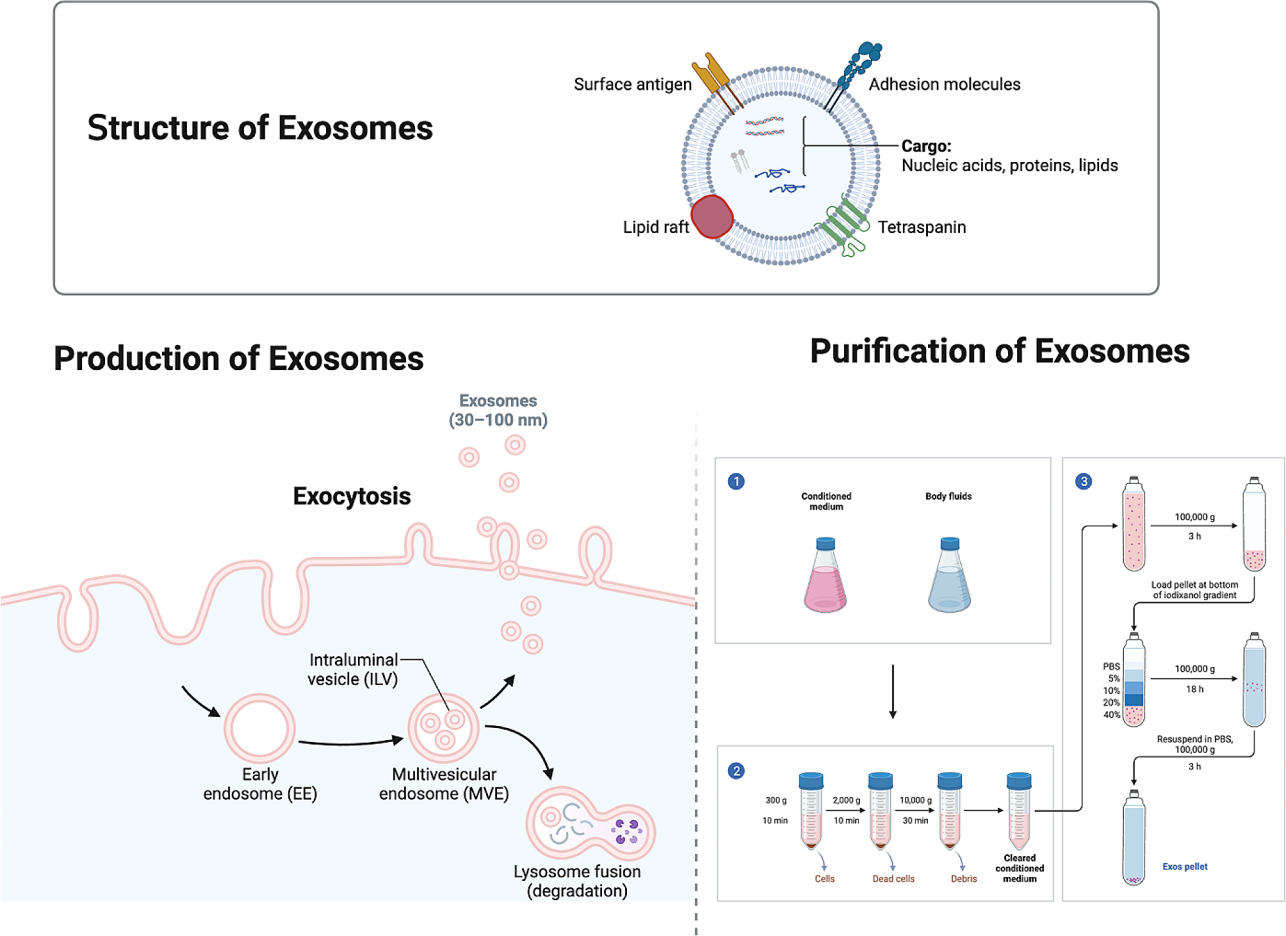
Structure, production, and purification of exosomes

Exosomes are tiny vesicles enclosed by a membrane, essential in the process of communication between cells. Found across all biological systems, cells secrete these exosomes under both regular and diseased states. Their sizes vary from 30 to 150 nm in diameter, and they originate from endosomes, following a distinct biogenesis route when compared to other extracellular structures like microvesicles and apoptotic bodies [21–24]. These vesicles exhibit a spherical configuration with a lipid bilayer and incorporate specific proteins, including CD63, CD81, CD9, flotillin, and ALIX (PDCD6-interacting protein), which are one of their identifying features [22, 25]. (Fig. 2)

**Fig. 3:**
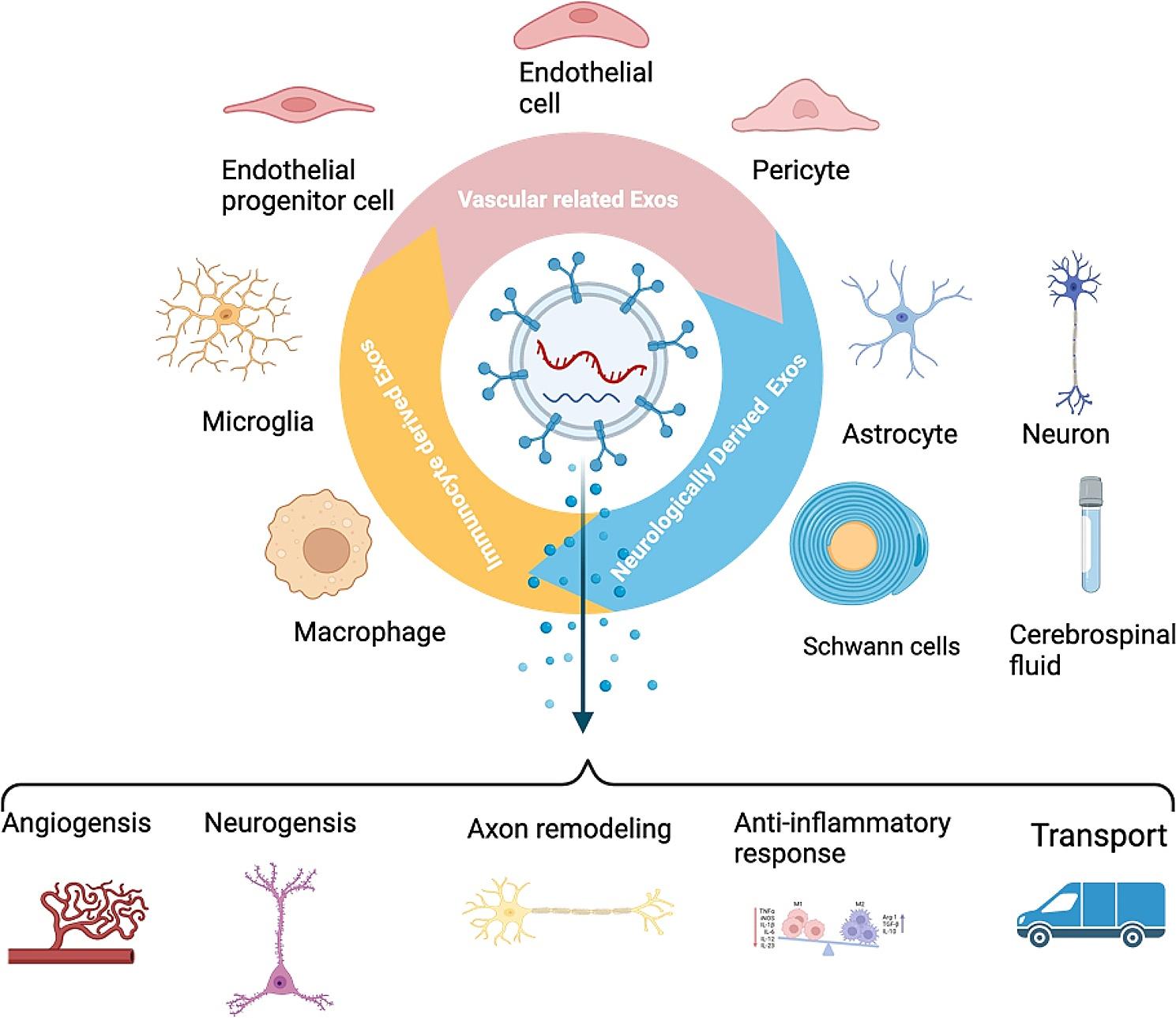
The multiple non-stem cell-derived exosomes and multimodal therapy strategies for neurotrauma therapy

Due to their potential to improve safety and therapeutic efficacy in different regenerative applications, exosomes have emerged as an alternative to cellular therapies. Most current exosome research centers on those derived from various stem cell sources. These studies often focus on modifications based on properties such as the regenerative abilities of the source cells. However, stem cell-related uncertainties and ethical issues limit this approach [25–27]. However, exosomes of specific cellular origin generally carry the properties of the source cells, are not ethically problematic, and are able to target the pathological processes that occur during neurotrauma [28–30]. Importantly, exosomes can carry proteins, lipids, and RNAs that transmit information between various cell types, affecting both normal physiological states and pathological states. Under normal physiological conditions, exosomes facilitate communication within the CNS and between the CNS and peripheral tissues. Oligodendrocytes contribute trophic support to axons, while neurons ensure the integrity of the BBB by delivering miR-132 to endothelial cells via exosomes [31–33]. Neurotraumatic events disrupt the BBB, enabling the passage of exosomes and various extracellular vesicles from injured or necrotic brain cells into the bloodstream and cerebrospinal fluid. These vesicles provoke inflammatory reactions in peripheral immune cells and stimulate microglia in the brain. Furthermore, exosomes originating from activated peripheral immune cells may penetrate the central nervous system, thereby amplifying the CNS’s response to the injury [32, 34–36]. Moreover, exosomes can transport cargo across the BBB and BSCB to reach distant organs without significant side effect [37–40] (Fig. 3).

This review classifies exosomes into three major categories according to their origin, neural, immune, and vascular and analyzes exosomes and their cargo as a therapeutic strategy for Neurotrauma, providing new strategies to prevent long-term disease progression. We will also discuss the prospects and challenges of non-stem cell-derived exosomes, as non-stem cell-derived exosomes may become a promising treatment method for neurotrauma in the future.

## Neurologically derived exosomes for Neurotrauma Therapy

This section explores the potential mechanisms and therapeutic effects of exosomes derived from neurogenic cells, fluids, or tissues, that have been developed to treat neurotrauma, including neurons, astrocytes, and Schwann cells, as well as exosomes from cerebrospinal fluid (CSF), the subventricular zone, and olfactory ensheathing cells. Studies on neurologically derived exosomes in neurotrauma, discussed in this section, are detailed in Table 1 with information such as the source of exosomes, route of administration, the animal model employed, exosomes modification and proposed mechanism.

**Table 1:** Neurologically derived exosomes for neurotrauma therapy

Study	Source of Exosomes	Animal	Trauma Model	Administration	Exosomes modification	Proposed mechanism	Refs
Xu, et al.	Neuron	Mouse	SCI	IV(Intravenous injection)	-	Reverse the activation of microglia and astrocytes and promote the maturation of OPCs	[45]
Jiang D, et al.	Neuron	Mouse	SCI	IV	miR-124-3p	Suppressing the activation of neurotoxic microglia and astrocytes	[46]
Zhang, et al.	Astrocyte	Mouse	TBI	IV	-	Suppressing mitochondrial oxidative stress and apoptosis	[55]
Lu, et al.	Astrocyte	Rat	SCI	IV	-	Alleviating fibrosis	[56]
Wang, et al.	Astrocyte	Mouse	TBI	IV	Bcl-2 and Bax shRNA plasmids	Reducing apoptosis	[57]
Feng, et al.	Astrocyte	Rat	TBI	IV	GJA1-20k	Promoting mitochondrial autophagy and ameliorate apoptosis	[58]
Chen, et al.	Astrocyte	Mouse	TBI	IV	GJA1-20k	Downregulating the apoptosis rate and upregulated mitochondrial function	[58]
Pan, et al.	Schwann cell	Mouse	SCI	IV	-	Inhibiting CSPGS deposition	[64]
Pan, et al.	Schwann cell	Rat	SCI	IV	-	increase autophagy and decrease apoptosis	[65]
Ren, et al.	Schwann cell	Rat	SCI	IV	MFG-E8	Attenuating inflammation	[66]
Huang, et al.	Schwann cell	Rat	SCI	IV	-	Promoting angiogenesis	[67]
Li, et al.	cerebrospinal fluid	Mouse	SCI	LI (Local Injection)	-	Promote vascular regeneration	[70]
Ibrahim, et al.	Subventricular zone	Rat	SCI	IV	-	Attenuating inflammation	[72]
Ijaz, et al.	Subventricular zone	Rat	SCI	IV	-	Attenuating inflammation	[73]
Tu, et al.	Olfactory ensheathing cells	Vitro	-	-	-	Enhance the viability of neural progenitor cells	[77]
Fan, et al.	Olfactory ensheathing cells	Rat	SCI	LI	-	Switching the phenotype of macrophages/microglia	[78]

### Neuron-derived exosomes (Neun-Exos)

Neurons are the quintessential cellular components of the CNS, playing a crucial role in its diverse functions. Serving as the primary conduits for both electrical and chemical signals, neurons are essential in the processing and transmission of information. This forms an intricate network foundational to all aspects of brain and spinal cord activity [41, 42]. Neurons orchestrate a range of processes from basic physiological responses to the nuanced complexities of human thought, emotion, and behavior. They also collaborate with a variety of other cells to fulfill their roles, Neuron sustain the equilibrium of the CNS by facilitating dialogues with neighboring astrocytes and microglia among other supportive glial cells [43, 44]. As a result, the exosomes originating from neurons are of particular interest, especially in the context of neurotrauma therapy, where Neun-Exos have emerged as a potential application.

Both Xu et al. and Jiang et al. discovered that Neun-Exos could counteract the activation of microglia and astrocytes, and further enhance the maturation process of oligodendrocyte progenitor cells (OPCs) in both living organisms and cultured cells [45, 46]. Moreover, exosomes originating from neurons were found to foster neurite extension and the differentiation of neural stem cells into neurons. This modification in the neuronal regulatory environment could play a vital role in enhancing spinal cord functionality in mice with SCI. In a separate observation, Jiang et al. identified that miR-124-3p, found in the cargo of neuron-derived exosomes, showed the highest enrichment in these vesicles. MYH9 was pinpointed as a target gene downstream of miR-124-3p, and the PI3K/AKT/NF-κB signaling pathway might play a part in the control of microglia through exosomal miR-124-3p. Also in Jiang’s study, SCI rats showed significant improvements in motor function within 28 days of treatment, outperforming controls on the BMS score, footprint, MEP test, and Louisville Swim Scale [46]. Therefore, Neun-Exos containing genetic materials such as utilization of miR-124-3p as a cell-free therapeutic approach holds significant promise for enhancing functional restoration following injuries to the CNS.

Having established the foundational role of neurons and the significance of Neun-Exos, it’s crucial to explore the other cellular contributors in this intricate network.

### Astrocyte-derived exosomes (AST-Exos)

Astrocytes, which are five times more numerous than neurons in the human brain, represent the most prevalent type of glial cells. Their importance in brain function extends to numerous roles, such as sustaining the BBB, managing synaptic connections, contributing to the recycling of neurotransmitters, and offering metabolic support to neural tissues. Additionally, they are responsible for controlling regional blood circulation and engaging in both the repair and scarring processes within the brain after different types of injuries [47–49]. Therefore, AST-Exos has become a focal point of research for scientists. AST-Exos are filled with various biological molecules secreted by astrocytes. They may serve as pathways for transporting Macromolecular substances to both neighboring and distant cells, leading to a broad spectrum of functional alterations in the cells receiving them. It has been suggested that AST-Exos can provide neuroprotective effects by controlling neural uptake, differentiation, and activity [50, 51]. Intriguingly, AST-Exos that are secreted under normal circumstances are recognized for their neurotrophic and neuroprotective benefits. Conversely, when astrocytes release Exos in adverse conditions, such as nutrient scarcity, oxidative stress, or inflammation, these exosomes can exert a protective effect on neurons, enhancing both neurite regeneration and growth [52–54]. Therefore, a large amount of AST-Exos isolated under normal physiological conditions is used in the treatment of neurotrauma.

In their investigation of the role of AST-Exos in treating rats with a TBI model, Zhang et al. determined that this treatment was effective in diminishing neurobehavioral deficits, cognitive dysfunction, and brain swelling in the affected rats. Furthermore, AST-Exos considerably lessened the loss and atrophy of neuronal cells in these TBI rats. The neuroprotective qualities of AST-Exos were exhibited through the elevation of antioxidant enzyme activities such as superoxide dismutase (SOD) and catalase (CAT), among others. This led to a marked reduction in oxidative stress and levels of mitochondrial H_2_O_2_ within the hippocampal neurons of the TBI rats, culminating in enhanced neuroprotection and a decrease in neuronal apoptosis [55]. Glial scar is a significant factor affecting healing in SCI, Lu et al. discovered that AST-Exos can be taken up by pericytes surrounding the injured spinal cord. This uptake inhibits the proliferation of pericytes, thereby reducing the formation of neural glial scars and promoting the recovery of limb function after SCI [56]. 

AST-Exos is also recognized as finely engineered exosomes, were the subject of an investigation by Wang et al. They studied modified primary astrocytes-derived exosomes containing plasmids that express B-cell lymphoma-2 (Bcl-2) and Bcl-2-associated X-protein (Bax) shRNA in TBI conditions. These modified exosomes helped mitigate the reduction of Mcl-1, XIAP, and surviving protein levels in the brain and decreased the release of cytochrome c from mitochondria into the cytosol post-TBI. Additionally, the altered exosomes eased impairments in the miniature excitatory postsynaptic current (mEPSC) and long-term potentiation (LTP) within the hippocampus of TBI-affected mice, leading to enhanced motor skills and cognitive functioning after the injury [57]. Meanwhile, AST-Exos was used as a transport carrier by Feng et al.transported gap junction alpha 1-20k (GJA1-20k)protein to TBI in rats, which identified the AST-Exos with GJA1-20k promoted autophagy inhibited cell apoptosis in the lesioned cortices’ tissues of experimental TBI rats [58].

While neurons and astrocytes provide a substantial understanding of the CNS’s cellular interplay, another cell type, the Schwann cells, offers a unique perspective, especially in peripheral nerve injuries.

### Schwann cells-derived exosomes (SCs-Exos)

Schwann cells constitute the major cells of the peripheral nervous system and are involved in a variety of important peripheral biological functions such as conduction of nerve impulses, participation in nerve growth and regeneration, nutrition of neurons, production of extracellular neuromodulators, regulation of motor nerve activity, and mediation of antigens [59–61]. While Schwann cells are native to the peripheral nervous system, they are among the most extensively researched cell types for their crucial role in promoting axon regrowth. Known as SCs, these neuroglial cells are responsible not just for steering axon regeneration and myelination within the peripheral nervous system (PNS), but they also serve a similar purpose when introduced into the spinal cord [60, 62, 63]. This also leads to a reasonable belief that SCs-Exos could be a potential therapeutic approach for neurotrauma.

Pan et al. found that post-treatment with SCs-Exos after intravenous injection promoted functional recovery in rats with SCI and demonstrated that SCs-Exos was able to increase Toll-like receptor 2 (TLR2)expression on astrocytes while decreasing the deposition of chondroitin sulfate proteoglycans(CSPGs), while SC-Exos was involved in the TLR2-activated NF-κB/PI3K signaling pathway, both of which are influential in SCI recovery key factors, which were further identified by Pan in the follow-up study [64, 65]. Furthermore, the utilization of the IKKβ inhibitor SC-514 served to confirm this signaling pathway. SCs-Exos have been shown to promote axonal protection following SCI by enhancing autophagy and decreasing apoptosis. These effects appear to be closely linked with the EGFR/Akt/mTOR signaling pathway [64].

In a separate study, two other key factors of neurotrauma, alleviating neuroinflammation and promoting angiogenesis, were also shown to be altered by SCs-Exos. SCs-Exos have the potential to lessen LPS-induced inflammation in Bone Marrow-derived Macrophages. They achieve this by hindering M1 polarization and encouraging M2 polarization via Milk fat globule-epidermal growth factor-factor 8 (MFG-E8), with the SOCS3/STAT3 signaling pathway playing a role in the process of M2 polarization [66].On the other hand, Huang et al. found in vitro and in vitro experiments that SCs-Exos can be taken up by vascular endothelial cells SCs-Exos promote angiogenesis by delivering integrin-β1 [67].

Given the multifaceted restorative role of SC-Exos may serve as a promising novel therapeutic agent for enhancing neurological functional recovery after neurotrauma.

### Cerebrospinal fluid-derived exosomes (CSF-Exos)

The cerebrospinal fluid (CSF) that envelops the brain and spinal cord is predominantly produced by the choroid plexus within the brain’s ventricles. CSF-Exos have been explored as potential diagnostic and prognostic markers for CNS disorders [68, 69]. Moreover, additional studies have demonstrated that externally introduced CSF-Exos may modify neurological functions following SCI. In particular, the study by Li et al. discovered that CSF-Exos loaded onto hydrogels could be absorbed by vascular endothelial cells. This uptake activated the PI3k/Akt signaling pathway, exerting angiogenic effects that may present new therapeutic possibilities for addressing acute SCI [70].

### Subventricular zone-derived exosomes (SVZ-Exos)

The subventricular zone (SVZ) of the lateral ventricles and the subgranular zone within the hippocampal dentate gyrus are identified as the primary sources of neural stem/progenitor cells (NS/PCs) in the mature brain [71].Mohammed et al. found improved motor function in rats by intrathecal injection of SVZ-Exos in the rat model of SCI, and the mechanism studied was mainly improvement of neuroinflammation at the site of spinal cord injury, The NOD-like receptor protein 3 (NLRP3) inflammatory complex formation, and in another of their study they reported that SVZ-Exos were able to significantly decreased the gene and protein expression of TNF-α,IL-1β,IL-18, and IL-6, prevented extensive tissue damage. Thus SVZ-Exos are therefore one of the candidates for the elimination of inflammation in neurotrauma [72, 73].

### Olfactory ensheathing cells derived exosomes (OECs-Exos)

Olfactory ensheathing cells (OECs) as a unique type of glial cells, are found in several regions of the olfactory system, including the lamina propria in the olfactory mucosa, the outer layer of the olfactory bulb, as well as both the inner and outer layers of nerve fibers. These cells envelop the unmyelinated primary olfactory axons and play a crucial role in neural repair. They achieve this by migrating and promoting the growth of olfactory sensory axons from the nasal epithelium towards the olfactory bulb [74, 75]. Many research studies have shown that OECs boost neural regeneration by encouraging axonal myelination, producing vital support factors for regrowing axons like exosomes, neurotrophic factors, and extracellular matrix (ECM) molecules, and managing the phagocytosis of cell debris and neuroinflammation [75, 76]. Tu and colleagues revealed that exosome-like vesicles from human Olfactory Ensheathing Cells (hOECs) or hOEC-derived EVs increased the growth of neural progenitor cells (NPCs) and reduced t-BHP-induced cell toxicity. Although they didn’t carry out in vivo experiments, their findings indicate that NPCs treated with OECs-Exos could have enhanced growth and differentiation abilities, paving the way for future research on the potential therapeutic role of OECs-Exos [77]. Fan et al. confirmed in a rat model of SCI that OECs-Exos can provide neuroprotective effects by changing the phenotype of Macrophages or microglial cells, thus promoting the recovery of motor function in rats with spinal cord injury. These in vitro studies found that OECs-Exos partially reversed LPS-induced neuroinflammation by inhibiting NF-κB and c-Jun signaling pathways in vitro, preventing neuronal death, and also proved that OECs-Exos is a potential choice for promoting the treatment of neurotrauma [78].

### Therapeutic implications of neurologically derived exosomes in neurotrauma interventions

Within the realm of neurotrauma therapy, this section delves into the therapeutic potential of exosomes emanating from neurogenic sources. Highlighted are the pivotal roles of Neun-Exos in the CNS, especially in intercellular communication and post-trauma cellular modulation. Findings from Xu et al. and Jiang et al. underscore the Neun-Exos’ capacity to mitigate adverse post-trauma cellular effects and promote neural differentiation. Additionally, the exosomes derived from various other neurological sources, including astrocytes and olfactory ensheathing cells, show promise in neuroprotective effects and promoting recovery in spinal cord injuries. As the exploration of these exosomes continues, they emerge not just as products of the CNS’s central cells, but also as potential game-changers in the therapeutic landscape of neurotrauma.

## Immunocyte-derived exosomes for neurotrauma therapy

Exosomes, derived from specific immune cells like microglia and Macrophages, have recently garnered significant attention in the field of neurotrauma therapy. Their unique properties and potential therapeutic implications are reshaping our understanding of treatment strategies. This section provides an in-depth exploration of immunocyte-derived exosomes and their role in neurotrauma. For a detailed overview of the studies discussed, refer to Table 2 which information such as the source of exosomes, route of administration, the animal model employed, exosome modification and proposed mechanism.

**Table 2:** Immunocyte-derived exosomes for neurotrauma therapy

Study	Source of Exosomes	Animal Model	Trauma Model	Administration	Exosomes modification	Proposed mechanism	Refs
Li, et al.	Microglia	Mouse	SCI	IV	mRNA-151-3p	Attenuating neuronal apoptosis	[81]
Li, et al.	Microglia	Mouse	TBI	IV	miR-214-3p	Inhibiting neuronal autophagy	[82]
Peng, et al.	Microglia	Mouse	SCI	IV	-	Inhibiting oxidative stress and promoting the survival and function of endothelia cells	[83]
Zhou, et al.	Microglia	Mouse	SCI	IV	miR-672-5p	Inhibiting and neuronal pyroptosis	[84]
Fan, et al.	Microglia	Rat	SCI	IV	Resveratrol	Activating autophagy and inhibiting apoptosis	[85]
Zhang, et al.	Peripheral Macrophage	Rat	SCI	IV	-	Activation of microglial autophagy and enhancing the polarization of anti-inflammatory type microglia	[89]
Luo, et al.	M2 Macrophage	Mouse	SCI	IV	OTULIN protein	Modulating vascular regeneration and neurological functional recovery	[90]
Huang, et al.	M2 Macrophage	Rat	SCI	IV	-	Improve angiogenesis	[91]
Zeng, et al.	M2 Macrophage	Rat	SCI	IV	IKVAV peptides	Reducing infiltration of Macrophages, downregulating pro-inflammatory factors	[92]
Gao,et al.	M2 Macrophage	Rat	SCI	IV	Berberine	Anti-inflammatory	[93]
Zhang,et al.	M2 Macrophage	Mouse	SCI	LI	Nerve growth factor (NGF), Curcumin (Cur)	Anti-inflammatory	[94]

### Microglia-derived exosomes (MG-Exos)

Microglia are integral components of the CNS, constituting 10–15% of all its cells. Functioning as intrinsic mononuclear phagocytes, they play a pivotal role in maintaining CNS homeostasis. Their involvement spans various processes, from axon expansion and synaptic adaptability to neuronal death under normal physiological conditions. Recent research has illuminated the significant role exosomes play in microglial function [79, 80]. Given this understanding, numerous studies are currently underway, exploring the potential of MG-Exos in neurotrauma therapy.

Research into exosomal microRNAs derived from microglia has unveiled some promising findings. For instance, microRNA-151-3p has been identified as having potent neuroprotective effects. This particular microRNA seems to mediate its effects by regulating neuronal apoptosis and axonal regeneration, primarily through the p53/p21/CDK1 signaling pathway. These findings have been validated in both living organisms (in vivo) and under laboratory conditions (in vitro) [81]. In a separate study, an intriguing observation was made regarding miR-124-3p in MG-Exos. Post-TBI, there was a noticeable increase of miR-124-3p in vitro. This elevation is believed to potentially impede neuronal autophagy, and by transferring to neurons, it may offer protection against neurological damage [82]. MG-Exos also plays an important role in one of the key factors of Neurotrauma repair, vascular repair. A study by Peng et al. found that MG-Exos, in both in vivo and in vitro studies, reduced the levels of oxidative stress (ROS), as well as NADPH oxidase 2 (Nox2) levels, and increased H_2_O_2_-induced bEnd.3 cell survival in vitro, tube formation and migration capacity, thereby promoting the survival and function of pinal microvascular endothelial cells (SCMECs), in which the keap1/Nrf2/HO-1 signaling pathway and downstream NQo1, Gclc, Cat and Gsx1 genes may be potential mechanisms to protect SCMECs from the toxic effects of oxidative stress and promote repair of SCI [83].

Similarly, MG-Exos is also a good carrier for transport. Zhou et al. found that miR-672-5p is the most critical miRNA associated with MG-Exos, and its target gene is Absent In Melanoma 2(AIM2). Using miR-672-5p-enriched MG-Exos can be inhibited by intravenous injection into spinal cord injured mice after which it was found to inhibit the activity of AIM2 to suppress AIM2/ASC/caspase-1 signaling pathway, thereby inhibiting neuronal scorching and ultimately promoting functional behavioral recovery in SCI mice [84]. MG-Exos can also transport small-molecule drugs. MG-Exos loaded with resveratrol are effective in crossing the BSCB, activating the PI3K signaling pathway, inducing autophagy, and inhibiting neuronal apoptosis. This facilitates the recovery of neural function in SD rats after SCI, improving paralysis of the lower limbs [85].

### Macrophage-derived exosomes (MAC-Exos)

Macrophages have a crucial function in neuroinflammation and tissue repair due to their capability to differentiate into two specific phenotypes: the “classically activated” M1 or the “alternatively activated” M2 phenotype. Increasing evidence shows that M1 Macrophages can produce significant amounts of pro-inflammatory cytokines, which can aggravate acute injuries. In contrast, the M2 phenotype is linked to a “restorative” role, aiding in tissue rejuvenation. Studies have revealed that exosomes derived from M2 Macrophages in injuries to the CNS can stimulate vascular renewal, cellular growth, and tissue expansion [86–88]. As a result, these exosomes are instrumental in aiding the recovery process in neurotrauma.

Zhang et al. investigated MAC-Exos and found that they could promote the polarization of anti-inflammatory microglia in vitro, thereby exerting anti-inflammatory effects. This is seen as playing a crucial role in the anti-inflammatory process. Further research revealed that MAC-Exos could suppress microglia autophagy by inhibiting the PI3K/AKT/mTOR autophagy signaling pathway. This inhibition may serve as a potential molecular mechanism for encouraging microglia polarization. In a rat model of SCI, an intravenous injection of MAC-Exos was observed to facilitate the recovery of motor function in the subjects [89] .

On the other hand, MAC-Exos also exemplified good vascular repair function, and Luo et al. explored the potential of MAC-Exos to stimulate the angiogenic activity of SCMECs, both in vitro and in vivo. In their investigation, they identified the ubiquitin thioesterase otulin (OTULIN) protein as a vital factor in fostering angiogenesis. The study revealed that MAC-Exos could activate the Wnt/β-catenin signaling pathway by transferring the OTULIN protein [90].Huang et al. found that MAC-Exos promotes angiogenesis and HIF-1/VEGF signaling pathway are also associated, and specific siRNAs to inhibit HIF-1α expression, reduced VEGF expression was observed, and tube formation, migration, and proliferation of bEnd.3 cells were attenuated in response to HIF-1α-siRNA treatment [91].

M2 MAC-Exos are also very well-engineered exosomes, and Zeng et al. designed a novel nanomagnet for SCI therapy using click chemistry to attach bioactive Ile-Lys-Val-Ala-Val (IKVAV peptides) to the surface of M2-Exos (MEXI). MEXI can inhibit Macrophages by reprogramming them to inflammation and promote neuronal differentiation of NSCs. In a mouse model of SCI, engineered exosomes were found to improve motor recovery in SCI mice by targeting Macrophage infiltration at injured sites in the spinal cord, downregulating pro-inflammatory factors, and improving regeneration of injured neural tissue after tail vein injection [92]. Gao et al. employed M2 MAC-Exos to create an innovative drug delivery system for Berberine, an established anti-inflammatory agent. Through comprehensive in vitro and in vivo experiments, the team showed that Berberine-infused exosomes (Exos-Ber) could reduce the M1 protein marker iNOS and augment the M2 protein marker CD206. Simultaneously, they noted a decrease in inflammatory and apoptotic cytokines, including TNF-α, IL-1β, IL-6, Caspase 9, and Caspase 8, underscoring the potent anti-inflammatory and anti-apoptotic abilities of Exos-Ber [93]. In a related effort, Zhang devised a parallel approach using M2-Exos, incorporating nerve growth factor (NGF) and curcumin (Cur). This resulted in the creation of stable engineered exosomes, approximately 120 nm in size, from primary M2 Macrophages. These specially designed exosomes, termed Cur@EVs-cl-NGF, exhibited anti-inflammatory and neuroprotective properties. Importantly, NGF is attached to the extracellular vehicles (EVs) using matrix metalloproteinase 9 (MMP9), a cleavable linker that is specifically released at the injury location. This method effectively curbs excessive inflammatory reactions and reduces subsequent damage to the spinal cord. Additionally, Cur@EVs-cl-NGF enables a timely dispersion of NGF into the surrounding microenvironment, offering protection against damage to neuronal cells [94].

### Therapeutic implications of immunocyte-derived exosomes in neurotrauma interventions

The therapeutic potential of immunocyte-derived exosomes in neurotrauma therapy is becoming increasingly evident. Microglia-derived exosomes, representing a significant portion of CNS cells, play pivotal roles in processes like axon expansion, synaptic adaptability, and neuronal health, with specific microRNAs like microRNA-151-3p and miR-124-3p showcasing neuroprotective effects. Similarly, MACrophage-derived exosomes, with their ability to differentiate into M1 and M2 phenotypes, have been shown to play crucial roles in both neuroinflammation and tissue repair. Studies have highlighted their ability to foster angiogenesis, transport therapeutic molecules, and influence key signaling pathways. Collectively, these findings underscore the transformative potential of immunocyte-derived exosomes in advancing neurotrauma treatments.

## Vascular-related exosomes and neurotrauma therapy

Neurotrauma directly leads to disruptions in the BBB and BSCB, compromising the microvasculature of the brain and spinal cord. This damage also extends to other barrier structures like pericytes [95, 96]. Given this, the potential therapeutic roles of vascular-derived exosomes have become a focal point of research. In this section, Extensive research has been conducted on exosomes derived from Endothelial Progenitor Cells, Endothelial Cells, Endothelial Colonies, and Pericytes. Table 3 with information such as the source of exosomes, route of administration, the animal model employed, exosomes modification and proposed mechanism.

**Table 3:** Vascular-related exosomes and neurotrauma therapy

Study	Source of Exosomes	Animal Model	Trauma Model	Administration	Exosomes modification	Proposed mechanism	Refs
Pan, et al.	Endothelial progenitor cells	Vitro	-	-	-	Ameliorating neuronal apoptosis	[100]
Ma, et al.	Endothelial progenitor cells	Vitro	-	-	miR − 210	Improving mitochondrial function	[101]
Yerrapragada et al.	Endothelial Progenitor cells	Vitro	-	-	miR − 210	Enhancing the protective effects on neuron apoptosis, oxidative stress, and decreased viability.	[102]
Li, et al.	Endothelial Progenitor cells	Vitro	-	-	miR-317	Boosts the neuroprotective effects of Againsting apoptosis and mitochondrial dysfunction	[103]
Ge, et al.	Microvascular endothelial cells	Mouse	SCI	IV	USP13	Regulating immune microenvironment	[106]
Gao, et al.	Endothelial colony-forming cell	Mouse	TBI	IV	-	Beneficial effects on BBB integrity	[107]
Yuan, et al.	Pericytes	Mouse	SCI	IV	-	Improve Microcirculation and Protect BBB	[110]

### Endothelial progenitor cell-derived exosomes (EPC-Exos)

Typically, endothelial progenitor cells (EPCs) reside primarily in the bone marrow. These cells, when signaled by chemokines, migrate from the bone marrow to injury sites, promoting repair. They not only differentiate to form new vascular networks but also release vital angiogenic growth factors and exosomes, fostering endogenous neurogenesis and functional recovery [97–99].

EPC-Exos have been shown to mitigate injury caused by hypoxia/reoxygenation (H/R) in both endothelial cells (ECs) and neurons. Pan et al. revealed that EPC-Exos not only reduced the apoptosis rate in neurons treated with oxygen-glucose deprivation (OGD) but also identified miR-221-3p as a biological mechanism for reducing OGD-induced neuronal apoptosis through the regulation of BCL2 Like 11 (BCL2L11) expression. This elucidates a potential new therapeutic strategy for cerebrovascular diseases [100].

Further studies highlight the role of miR-210-enriched EPC-Exos in enhancing this protective effect. They promote ECs proliferation and migration, mildly improve mitochondrial function by correcting mitofusin 2(mfn2) and dynamin-related protein-1(drp1) dysregulation, and ameliorate H/R injury in neurons [101]. Yerrapragada et al. demonstrated that EPC-Exos enriched with miR-210 significantly enhanced cell viability and reduced both neuronal apoptosis and ROS production when compared to EPC-Exos without miR-210. Their findings also revealed that miR-210 was released and transferred to neurons in a manner that was dependent on time. Overall, the loading of miR-210 appears to enhance the protective effects of EPC-Exos against H/R-induced injuries in both ECs and neurons [102].

Separately, Li et al. reported that EPC-Exos with high miR-137 expression mitigated apoptosis, mitochondrial dysfunction, and ferritinization, and inhibited iron toxicity in SH-SY5Y cells exposed to oxyhemoglobin (oxyHb) in vitro. The underlying protective mechanism may involve the miR-137-COX2/PGE2 signaling pathway [103].

### Endothelial cell-derived exosomes (ECs-Exos)

Endothelial cells (ECs) are fundamental elements in the neurovascular unit (NVU) and are essential to CNS homeostasis and function. These cells are responsible for maintaining the balance of the CNS microenvironment, a role they fulfill by creating the BBB, facilitating precise interactions within the NVUs, and controlling the transportation of nutrients and large molecules. Exosomes stand out as one of the key means of intercellular communication within NVUs [104, 105].

In the context of neurotrauma, there’s a predominant differentiation of Macrophages into the M1 phenotype. These cells infiltrate the spinal cord or brain through the compromised BSCB or BBB. However, the presence of the M2 phenotype is only transient. A study by Ge et al. showcased how the ECM, combined with ECs-Exos, could steer microglia/Macrophages toward M2 polarization, offering therapeutic potential. This shift was found to reduce mitochondrial damage and decrease the production of ROS. These effects not only facilitated motor recovery but also promoted M2 polarization of microglia/Macrophages, an outcome that the researchers believe may be mediated by ubiquitin-specific protease 13 [106].

### Endothelial colony-forming cell-derived exosomes (ECFC-Exos)

Gao and colleagues discovered that pre-treatment with ECFC-exos led to an increase in the migration of scratched endothelial cells (ECs). When ECs exposed to hypoxia were incubated with these ECFC-Exos, there was a reduction in PTEN expression, an enhancement in AKT phosphorylation, and an increase in the expression of tight junction (TJ) proteins within the cells. In an animal model examining TBI, the ECFC-Exos enhanced the Evans Blue (EB) dye extravasation, reduced brain swelling, and decreased TJ degradation. These exosomes also contributed to the integrity of the BBB in TBI-affected mice and facilitated the recovery of motor function following TBI [107].

### Pericytes derived exosomes (PER-Exos)

Pericytes, which envelop endothelial cells in capillaries and veins across the body, are vital components of the NVU. Their multifaceted roles range from regulating microcirculatory pressure and maintaining microvascular integrity to controlling permeability and angiogenesis. These functions are mediated through direct interactions and paracrine signaling mechanisms [108, 109].

Yuan and colleagues conducted a study where they transplanted PER-Exos into mice with SCI. These PER-Exos were found to alleviate pathological alterations in the affected spinal cord and enhance motor function, blood circulation, and oxygen levels post-SCI. Moreover, PER-Exos was shown to augment the endothelial cells’ capacity to manage blood flow, fortify the BBB, diminish swelling, and suppress endothelial cell death. This mechanism was found to be linked to the PTEN/AKT pathway. The unique connection between pericytes and endothelial cells enables the latter to absorb exosomes from pericytes, thus playing a role in modulating endothelial function, so the repairing effect of exogenous pericyte-derived exosomes on endothelial cells as well as neurovascular units and BBB or BSCB is of interest [110].

### Therapeutic implications of vascular-derived exosomes in neurotrauma interventions

Vascular-derived exosomes are emerging as influential players in neurotrauma therapy, particularly in addressing the challenges posed by disruptions to the BBB and BSCB. Exosomes from various vascular sources, including EPCs, ECs, ECFCs, and pericytes, each present unique therapeutic potentials. EPC-derived exosomes, for instance, have shown promise in promoting angiogenesis and neurogenesis, with specific microRNAs playing pivotal roles in neuroprotection. Meanwhile, exosomes from ECs are crucial for maintaining the CNS microenvironment, with potential in guiding beneficial Macrophage polarization. ECFC-derived exosomes demonstrate protective effects on the BBB, while pericyte-derived exosomes play a role in maintaining vascular integrity and function, especially post-neurotrauma. Collectively, these findings underscore the profound therapeutic potential of vascular-related exosomes in neurotrauma interventions.

## Challenges and future perspectives

Extracellular vesicles (EVs) mainly encompass both exosomes and microvesicles, yet they exhibit distinct structural and functional characteristics. Exosomes typically range from 30 to 150 nm in diameter, enabling them to traverse stringent cellular barriers such as the BBB more effectively than the larger microvesicles, which measure between 100 and 1000 nm [111, 112]. Originating from multivesicular bodies (MVBs), exosomes are released into the extracellular environment through the fusion of MVBs with the plasma membrane. This release mechanism positions exosomes as crucial facilitators of intercellular communication. They are particularly adept at transporting specific therapeutic agents, including small RNAs and proteins. Furthermore, exosomes are less immunogenic and more biocompatible than microvesicles, which are generated by the outward budding and subsequent cleavage of the cell membrane. This biocompatibility, along with their cargo-delivery efficiency, renders exosomes as promising candidates for therapeutic application [24, 113, 114].

While exosomes from mesenchymal stem cells have garnered attention, exosomes derived from non-stem cells present unique therapeutic opportunities. These exosomes, thanks to their innate ability to target damaged tissues, play a pivotal role in neurological restoration. Their small molecular dimensions, innate molecular transport attributes, and agreeable biocompatibility have positioned exosomes as valuable prospects for drug delivery in contemporary medicine [115, 116]. Conventional medicines frequently encounter obstacles that hinder their effectiveness and clinical application. These challenges include poor water solubility, rapid body elimination, less-than-ideal biocompatibility, insufficient distribution within the body, and reduced cellular permeability [29, 117, 118]. Concurrently, there is a burgeoning development of novel engineered vesicles. Examples include liposomes, polymeric nanoparticles, and solid lipid nanoparticles (SLNs), which have been enhanced in terms of their biological performance through surface modifications and the alteration of their cargo [119–121]. Nevertheless, these engineered vesicles must still overcome certain challenges to further advance the field. A primary obstacle is the innate barrier of the nervous system, the BBB which engineered vesicles must penetrate. Although some progress has been made by modifying their surfaces with cell-penetrating peptides (CPPs), the efficacy of these modifications remains limited. A second consideration is biocompatibility and immunogenicity [122]. Despite improvements in reducing the biocompatibility and immunogenicity of new engineered vesicles, they still do not compare favorably with exosomes. Exosomes merge the benefits of both cellular and nanotechnological approaches in drug administration. For instance, they enhance drug stability and naturally target specific areas based on donor cells during delivery. Being nanoscale molecules composed of cell surface material, exosomes also exhibit robust biological barrier permeability, allowing them to selectively access tissue damage [115–117]. We propose the hypothesis that non-stem cell-derived exosomes could emerge as a significant therapeutic agent in the treatment of neurotrauma, and additionally, as a promising system for drug delivery. The varied sources of non-stem cell-derived exosomes add to their appeal, and in this review, we delve into the functions of exosomes originating from multiple cellular or humoral sources in the context of neurotrauma. The diversity of sources also brings about a diversity of repair mechanisms, offering different therapeutic possibilities for complex pathological changes in neurotrauma, which offers more advantages than the singularity of stem cell-derived exosomes, while being more accessible and freer of ethical issues than stem cells or stem cell-derived exosomes.

While non-stem cell-derived exosomes offer promising avenues in neurotrauma therapy, several challenges remain. To address these, the International Society for Extracellular Vesicles updated the Minimal Information for Studies of Extracellular Vesicles (MISEV) guidelines to guide exosome preparation and characterization. MISEV2018 categorizes information as “required,” “should be provided if possible,” or as an “alternative if all recommendations cannot be fully adhered to.” A key aspect is the need to standardize extracellular vesicle (EV) production and quality control to facilitate clinical translation [111].

Recent developments in clinical-grade exosome production have been reported, including the work of Mayela Mendt et al., who described a bioreactor-based, large-scale production of clinical-grade exosomes adhering to Good Manufacturing Practice (GMP) standards [123]. Although there are various methods to augment the production of exosomes, such as engineering modifications to exosomes and stimulation with exogenous drugs, enhancing exosome yield at the source remains a principal strategy [124]. Secondly, the selection of isolation approaches to facilitate exosome production also presents challenges, with each method having its advantages and drawbacks. For instance, differential ultracentrifugation, the most widely used method, involves sequential centrifugation steps at varying speeds to eliminate cells and debris, followed by a final ultracentrifugation step at high speed to concentrate the exosomes. Density gradient centrifugation, on the other hand, involves layering the sample onto a density gradient (typically sucrose or iodixanol) after initial centrifugation steps, and then ultracentrifuging it. Exosomes are then isolated from a specific layer of the gradient based on their buoyant density. Additionally, size-exclusion chromatography (SEC) offers high-quality exosome preparation with excellent reproducibility. It is particularly suited for high-throughput industrial applications, given that the gravity flow used in SEC minimizes damage to exosome structure and function [111, 125].

For exosomes that meet clinical-grade standards, establishing uniform characterization standards is crucial. Characterization encompasses the four types of extracellular vesicle (EV) characterization delineated in MISEV2018, namely: (1) quantitative, (2) qualitative, (3) single vesicle, and (4) topology characterization. Quantitative characterization involves assessing the quantity and concentration of exosomes, commonly employing methods such as nanoparticle tracking analysis or protein quantitation techniques. Qualitative characterization focuses on identifying and confirming specific exosomal markers, like membrane proteins and vesicular contents. Single vesicle characterization is dedicated to examining individual exosome attributes, including size, shape, and surface properties, using techniques like Transmission Electron Microscopy (TEM) or Atomic Force Microscopy (AFM). Topology characterization, on the other hand, seeks to elucidate the physical and chemical attributes of exosomes, such as lipid composition and membrane bilayer structure. These comprehensive methods are vital in ensuring the uniformity and functional integrity of exosomes for clinical use [111, 125, 126].

There is a need to delve deeper into the content of exosomes to discern which elements may be beneficial for treating neurotrauma and which might be detrimental. Additional investigation is required to explore the correlation between injection frequency and dosage, and the sustainability of the long-term effectiveness of exosomes from various origins. Understanding whether single or multiple doses could lead to adverse consequences is also vital for the appropriate utilization of exosomes in neurotrauma therapy. In addition, studies for the treatment of neurotrauma with non-stem cell-derived exosomes are still at a stage and most of them are based on rodents, especially C57 mice and SD rats. While there exist anatomical distinctions between human and rodent CNS, with rodents typically having a smaller zone of neurological injury and humans having a more extensive one, leading to greater tissue damage, the disparities go beyond that. The human nervous system’s complexity and advancement surpass that of rodents, and the way human Neurotrauma unfolds through immune, vascular, and inflammatory responses, as well as glial scar formation, diverges significantly from rodent neurotrauma [8, 127, 128]. Consequently, there is a need to broaden research to include larger animals like monkeys in preclinical studies. Even though exosomes have demonstrated clear therapeutic benefits in treating neurotrauma, the precise therapeutic mechanisms and targets remain incompletely explored, and most investigations have emphasized the impacts of miRNAs, leaving other exosomal components less studied. Thus, additional research is essential to elucidate the therapeutic potential of exosomes. Furthermore, there’s a deficit in cross-sectional comparative research concerning exosomes from varied non-stem cell origins in Neurotrauma, and the distinctions in the effectiveness of different exosomes are still ambiguous.

## Conclusion

In summary, addressing neurotrauma effectively remains elusive due to the intricate pathophysiological changes it induces and the lack of robust treatments. The limited regenerative capacity of the CNS further compounds the challenge. However, exosomes, especially those from non-stem cell sources, are emerging as potential therapeutic agents, capable of navigating barriers like the BBB or BSCB and become therapeutic drugs and can also serve as good drug carriers to deliver small molecule drugs, proteins, or non-coding RNAs, which have great therapeutic potential for neurotrauma. Undoubtedly, there is a need to refine non-stem cell-derived exosomes by analyzing the variations in therapeutic targets and efficacy among exosomes from diverse sources. This will enhance their therapeutic impact in neurotrauma. Furthermore, additional research is essential to delineate precise tissue repair mechanisms. By addressing these questions, a solid theoretical foundation can be laid for the clinical application of non-stem cell-derived exosomes, bringing new hope to the clinical management of neurotrauma.
